# 360° Camera Versus Two-View Standard Video Camera Capture of Neonatal Resuscitation: Optimizing Recording for Observation of Resuscitation Steps

**DOI:** 10.7759/cureus.81625

**Published:** 2025-04-02

**Authors:** Nahee Park, Meghan Rowe, Megan M Gray, Ellie Ficco, Taylor Sawyer, Rachel A Umoren

**Affiliations:** 1 Department of Pediatrics, Division of Neonatology, University of Washington School of Medicine, Seattle, USA

**Keywords:** 360-degree video, medical education, neonatology nrp neonatal resuscitation, simulation debriefing, simulation education, simulation in medical education, video debriefing, video feedback

## Abstract

Background

Fixed-camera video recordings of neonatal resuscitation have been utilized to facilitate post-event debriefing and evaluate guideline adherence. Traditional video capture is limited in the field of view it provides, requiring multiple cameras to capture the relevant steps of the resuscitation.

Objective

We sought to examine the feasibility of a 360° video camera (360V) (Gear 360, Samsung, Vietnam) compared to a traditional two-view standard video camera (SVC) (SimView Mobile Camera, Laerdal Medical, New York) as a method for recording neonatal resuscitation and capturing salient resuscitation factors.

Methods

This observational study recorded neonatal resuscitation events in a simulated delivery room. Each simulation was recorded by two methods: near- and far-view SVC and 360V. Two independent reviewers analyzed the videos for visibility of Neonatal Resuscitation Program (NRP) steps, audibility, and recording quality of predefined resuscitation events and behaviors. Global visualization and audibility scores were analyzed using a five-point Likert scale.

Results

A total of 25 simulated neonatal resuscitation events were reviewed. The average global visualization of NRP steps was higher using 360V than with two-view SVC (4.26 vs. 3.33, P = 0.0001). Average global audibility using 360V was also higher than with two-view SVC (4.90 vs. 4.21, P < 0.0001). Motion sickness more often occurred while viewing 360V videos compared to two-view SVC videos (P < 0.0001, Fisher’s exact test). However, motion sickness was overall a rare occurrence.

Conclusions

Average global visualization and audibility were improved using 360V compared to two-view SVC. A 360V is a superior method for reviewing neonatal resuscitation simulations compared to two-view SVC. A limitation of the study is that the location of the SVC in the room may not reflect the ideal placement for capturing visual aspects of neonatal resuscitation. Placement of the camera on the warmer may have yielded different results. Further studies comparing various SVC placements to 360V are warranted.

## Introduction

Approximately 10% of all newborns require resuscitation with positive pressure ventilation, and approximately 0.1% require cardiac compressions and medications [1]. Neonatal resuscitation is a complex task requiring a high degree of cognitive, technical, and behavioral skill. Due to its complexity, errors frequently occur during neonatal resuscitation, and adherence to resuscitation guidelines is often poor. Prior studies have documented error rates as high as 23%, with common errors including overly aggressive stimulation, failure to assess heart rate and/or breath sounds, inadequate positive pressure ventilation, improper chest compression technique, and asynchronous positive pressure ventilation and chest compressions [2,3]. In one study, 54% of resuscitations had deviations from the Neonatal Resuscitation Program (NRP) guidelines [3]. The frequency of errors observed during neonatal resuscitations indicates that strategies are required to improve resuscitation performance.

Post-event debriefing is a proven strategy for improving the execution of neonatal resuscitations. A meta-analysis found that effective debriefings can improve individual and team performance by 25% [4]. Studies have shown that post-event debriefing of neonatal resuscitation is associated with improvements in both technical and behavioral skills [5-8]. The American Heart Association (AHA) also endorses post-event debriefing to improve cardiopulmonary resuscitation quality [9]. Incorporating video recordings into post-event debriefing may improve the quality of the debriefing [10-12]. A study on the effectiveness of video-enhanced debriefing by Nadler et al. showed that neonatal resuscitation teams had a significant improvement in teamwork scores over a four-month period following the start of video-enhanced debriefing [12].

Video recording in the delivery room is logistically challenging due to privacy concerns, policy barriers, space constraints, differences in lighting and angles, and the potential for technical issues. The typical video setup includes two or more SVCs positioned in strategic locations within the delivery room, typically above the warmer and on the ceiling [12]. Multiple cameras are required to get simultaneous video views of the infant on the warmer, as well as a wide-angle view of the resuscitation team. Incorporating multiple cameras and audiovisual recording equipment can be more expensive and may require more technical expertise. For example, the two-view cameras (SimView Mobile Camera, Laerdal Medical, New York) used in this pilot study cost roughly $7,700 [13]. The use of a single camera that allows a 360° view may overcome some of these obstacles. The 360° camera allows the recording of a full view of a room by using two 180° fisheye lenses mounted back-to-back [14]. The 360° video camera (Gear 360, Samsung, Vietnam) used in this study cost $199 [15]. During video review, the reviewer can pan across the footage and zoom in on areas of interest. Incorporating a single 360° camera in a delivery room may provide an easy and cost-effective method to record neonatal resuscitation for post-event video-enhanced debriefing.

In this pilot study, we compared the video capture of simulated neonatal resuscitation events using a two-view SVC to a 360° video camera (360V). We hypothesized that using a 360V device improves the visibility and audibility of neonatal resuscitation steps compared to using SVC. This article was previously presented as a meeting abstract at the 23rd Annual International Meeting on Simulation in Healthcare on January 21-25, 2023 [16].

## Materials and methods

Study design

The study followed an observational cohort design. Simultaneous video recordings of simulated neonatal resuscitations were performed using two methods: two-view SVC and 360V. Video and audio recordings from each method were then compared. The primary outcome was the average global visualization and global audibility scores for each video method [16]. Secondary outcomes included the proportion of resuscitation factors that were well visualized and the proportion that were clearly heard. This project was approved by the Seattle Children's Hospital Institutional Review Board under approval number STUDY00000298.

Setting and participants

For this pilot study, we conducted 25 simulated neonatal resuscitations at the University of Washington School of Medicine, Division of Neonatology, Neonatal Education and Simulation-Based Training (NEST) Laboratory from January through December 2017 [16]. All events simulated a bradycardic and hypoxic newborn requiring neonatal resuscitation with positive pressure ventilation, endotracheal intubation, chest compressions, and intravenous epinephrine administration [16]. The neonatal resuscitation team leader was a physician or nurse with Neonatal Resuscitation Program provider certification. Two embedded team members among the authors (MR and RU) were assigned as the bedside nurse and respiratory therapist. All simulations used a high-fidelity SimNewB® neonatal simulation mannequin (Laerdal Medical, Wappingers Falls, New York), positioned on a neonatal radiant warmer and operated by a simulation technologist. Standard neonatal resuscitation equipment and medications were available for each resuscitation.

**Figure 1:**
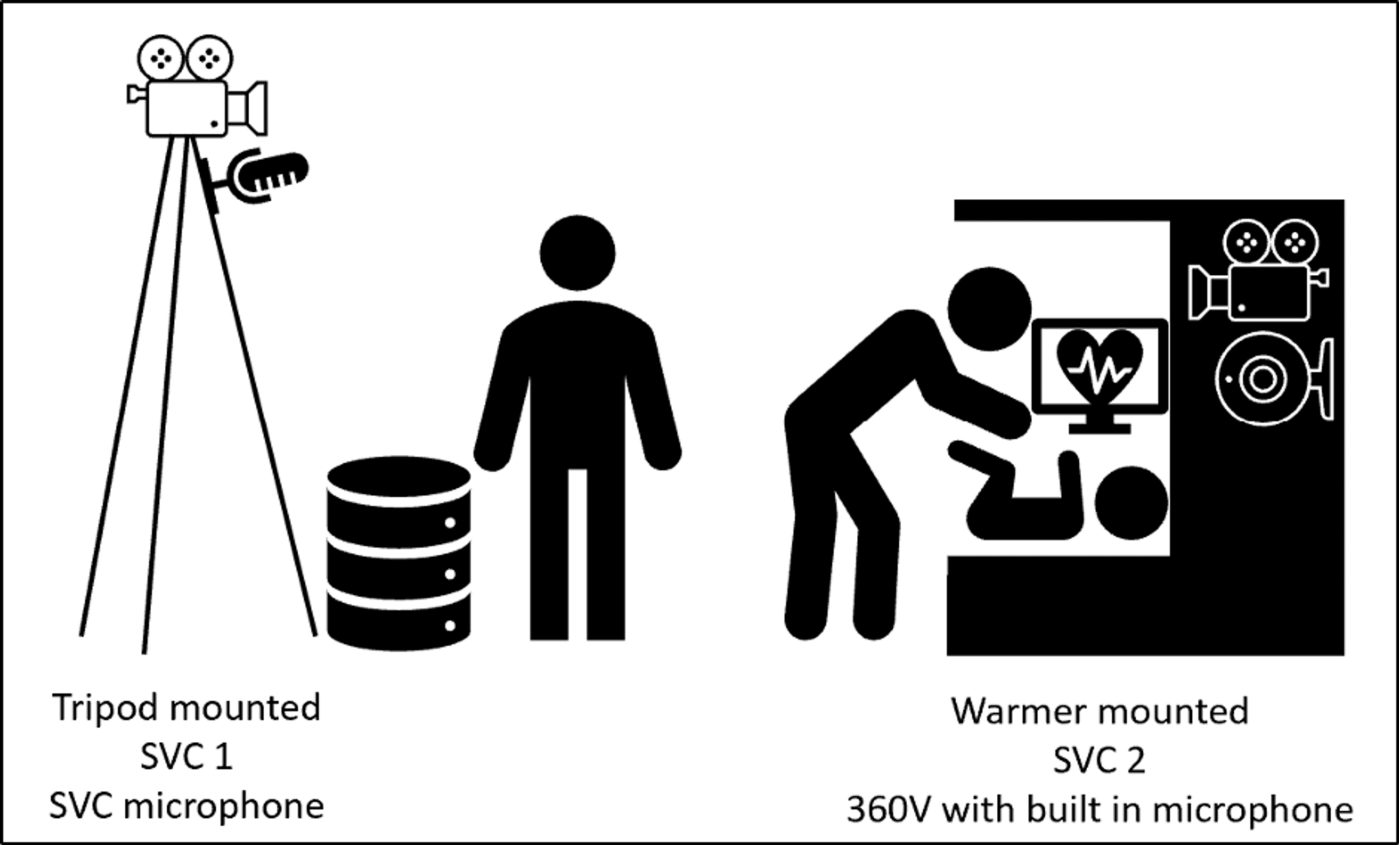
Camera and microphone locations 360V, 360° video camera; SVC, standard video camera. Image credits: Megan Gray, MD

All simulations were recorded simultaneously using two methods: (1) two-view SVC recording and (2) 360° video camera (360V) recording. The first SVC (SVC 1) and microphone were placed atop a tripod angled toward the resuscitation team, ensuring that the warmer bed, monitor, and equipment cart were in view (Figure 1). The second SVC (SVC 2) was mounted on the radiant warmer bed. The 360V, including its built-in microphone, was also mounted on the radiant warmer bed. The location of all recording equipment remained the same for all simulations. SVC videos were recorded at 480 pixels, while 360V videos were recorded at 4000 pixels.

Video evaluation

To assess neonatal resuscitation behaviors, 24 distinct factors were identified based on national guidelines, previous research on resuscitation quality, and expert consensus [1]. Two reviewers among the authors with neonatal resuscitation expertise scored all video recordings. Reviewers received detailed training with viewing instructions and completed a practice review before analyzing the simulations. Video reviewer responses were categorized into six levels based on how well each resuscitation factor was visualized or heard: (1) Excellent; (2) Good; (3) Fair; (4) Poor; (5) Bad; (6) N/A - not done in the scenario. Events categorized as "N/A - not done in the scenario" were excluded, as neither SVC nor 360V could capture an event that did not occur. For example, if intubation was not performed, this factor would not be included in event analyses. For SVC videos, reviewers noted which view (far camera on a tripod or near camera on the warmer) was used to visualize the factor.

Statistical analysis

The percentage of events that were well visualized for each resuscitation factor was calculated based on the total number of video reviews for each recording method. Differences in visualization and audio quality between recording methods were analyzed using Pearson’s chi-square test. Inter-rater reliability was assessed using the percent agreement and Gwet’s AC1 statistic, based on resuscitation factors within each observation method. Global visualization and audibility scores were analyzed using the original five-point Likert scale [16]. All analyses were conducted using Stata 18.0 (StataCorp LLC, Texas). A P-value of less than 0.05 was considered statistically significant.

## Results

A total of 25 resuscitation simulations were reviewed. Two experts reviewed all SVC and 360V recordings for all 25 events (50 total video reviews, 25 for each camera method). Of the 24 identified resuscitation factors, 15 (63%) were visualized or heard in all simulations when reviewing both SVC and 360V videos. Factors not performed or unable to be visualized or heard in all resuscitation events included chest rise with positive pressure ventilation (PPV), endotracheal intubation, airway confirmation using end-tidal carbon dioxide (ETCO₂), auscultation with stethoscope, rate of ventilation via endotracheal tube (ETT), chest compressions performed, depth of chest compressions, rate of chest compressions, code leader self-identification as code leader, and announcement of role assignments.

Average global visualization of NRP steps was higher using 360V than with two-view SVC (4.26 vs. 3.33, P < 0.001) (Table 1). There was improved visibility or audibility of statistical significance in 19 of the 24 (79%) identified resuscitation factors in 360V videos compared to SVC videos (Table 1, Figure 1). Tasks with improved visibility using the 360V versus two-view SVC cameras, based on median Likert scores and interquartile ranges (25th-75th percentile), included PPV mask ventilation, chest rise with PPV, quality of mask PPV, rate of mask PPV, endotracheal intubation, auscultation with stethoscope, rate of ventilation via ETT, intravenous (IV) placement, chest compressions, depth of chest compressions, rate of chest compressions, medications delivered, equipment function, and enactment of team roles. Average global audibility using 360V was higher than with two-view SVC (4.90 vs. 4.21, P < 0.0001) (Table 1). Audibility was superior in 360V compared to two-view SVC for verbal communication between team members, closed-loop communication, the ability to distinguish who is speaking, and clarity while multiple team members were speaking.

**Table 1:** Analysis of resuscitation factors identified in 360V videos versus SVC videos 360V, 360° video camera; SVC, standard video camera; ETCO_2_, end-tidal CO_2_; IV, intravenous, PPV, positive pressure ventilation; UVC, umbilical venous catheter; ETT, endotracheal tube. *Median Likert (25-75 intraquartile range). **Fisher's exact P-value.

Observations	N, SVC video with element seen	N, 360V video with element seen	SVC video*, N=25	360V video*, N=25	P-value**
Visualized element					
Set up of equipment	25	25	4 (4,4)	4 (4,4)	0.24
PPV mask ventilation	25	25	4 (1,5)	5 (5,5)	<0.0001
Chest rise with PPV	24	25	1 (1,1)	2 (1,3)	0.002
Quality of mask PPV	25	25	4 (1,4)	5 (4,5)	<0.0001
Rate of mask PPV	25	25	5 (1,5)	5 (5,5)	0.002
Endotracheal intubation	24	24	4 (2,4)	4 (4,5)	0.019
Airway confirmation (ETCO_2_)	13	12	1 (0,3)	0 (0,3)	1
Auscultation with a stethoscope	24	24	5 (4,5)	5 (5,5)	0.012
Rate of ventilation via ETT	24	24	5 (3, 5)	5 (4,5)	0.016
IV placement (UVC)	25	25	4 (1,5)	5 (5,5)	0.002
Chest compression	23	23	5 (1,5)	5 (5,5)	0.002
Depth of chest compression	23	23	4 (1,4)	4 (4,5)	<0.0001
Rate of chest compressions	23	23	5 (2,5)	5 (4,5)	0.024
Medications delivered	25	25	3 (2,4)	4 (4,4)	<0.0001
Equipment presence	25	25	5 (4,5)	5 (5,5)	0.081
Equipment function	25	25	4 (4,4)	5 (5,5)	<0.0001
Enactment of team roles	25	25	5 (4,5)	5 (5,5)	<0.0001
Heard elements					
Code leader self-identification as a code leader	9	11	5 (4,5)	5 (5,5)	0.068
Announcement of role assignments	16	12	5 (5,5)	5 (5,5)	1
Verbal communication between team members	25	25	4 (4,5)	5 (5,5)	<0.0001
Verbal communication of the leader	25	25	5 (4,5)	5 (5,5)	0.074
Closed-loop communication	25	25	4 (4,4)	5 (5,5)	<0.0001
Ability to distinguish who is speaking	25	25	4 (4,5)	5 (5,5)	<0.0001
Clarity while multiple people are speaking	25	25	3 (3,4)	5 (5,5)	<0.0001

**Figure 2:**
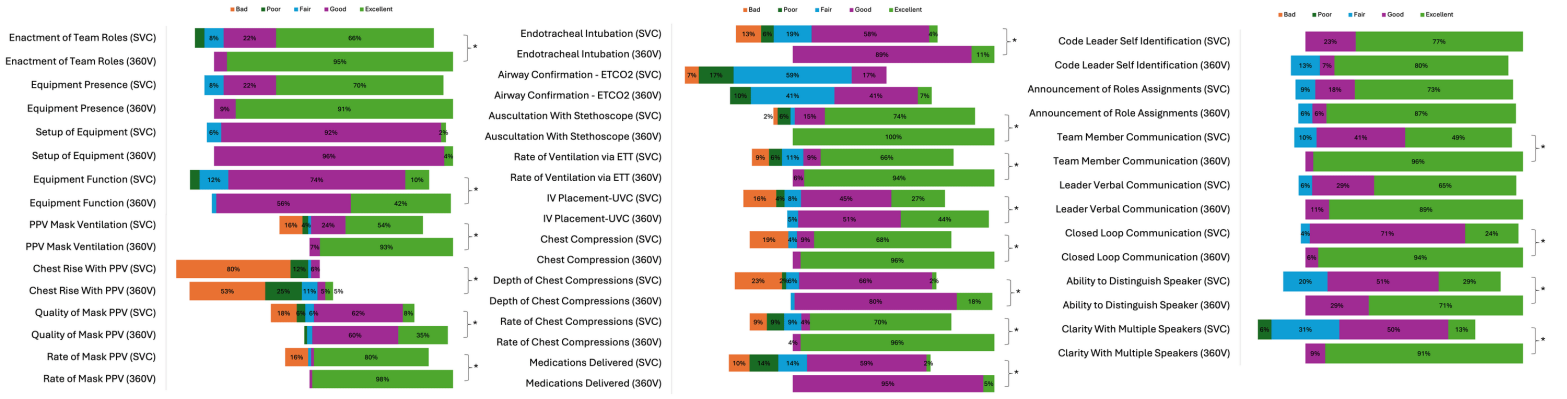
Reviewers’ analysis of 24 resuscitation factors via the following scale: 1, excellent; 2, good; 3, fair; 4, poor; 5, bad; and 6, N/A - not done in the scenario *P<0.05. ETT, endotracheal tube; UVC, umbilical venous catheter; PPV, positive pressure ventilation; 360V, 360° video camera; SVC, standard video camera.

Motion sickness was reported rarely or never with SVC videos and rarely or sometimes with 360V videos (P < 0.0001) [16]. Video reviewers demonstrated good consistency in evaluating the performance of identified resuscitation factors. The percentage agreement of event scoring had a median of 71% (IQR 58%, 82%), with the majority of events showing at least moderate agreement (88.5% with agreement >40%) (Table 2).

**Table 2:** Inter-rater agreement on observed elements ETCO_2_, end-tidal CO_2_; IV, intravenous; PPV, positive pressure ventilation; UVC, umbilical venous catheter; ETT, endotracheal tube.

Observations	Percentage agreement between raters	Gwet's AC1
Set up of equipment	0.89	0.88
PPV mask ventilation	0.67	0.64
Chest rise with PPV	0.33	0.25
Quality of mask PPV	0.4	0.32
Rate of mask PPV	0.96	0.95
Endotracheal intubation	0.62	0.59
Airway confirmation (ETCO_2_)	0.56	0.48
Auscultation with a stethoscope	0.89	0.88
Rate of ventilation via ETT	0.87	0.86
IV placement (UVC)	0.16	0.05
Chest compression	0.82	0.81
Depth of chest compression	0.71	0.68
Rate of chest compressions	0.84	0.83
Medications delivered	0.8	0.78
Equipment presence	0.8	0.78
Equipment function	0.36	0.24
Enactment of team roles	0.76	0.72
Code leader self-identification as a code leader	0.62	0.56
Announcement of role assignments	0.82	0.78
Verbal communication between team members	0.71	0.65
Verbal communication of the leader	0.69	0.62
Closed-loop communication	0.7	0.65
Ability to distinguish who is speaking	0.56	0.39
Clarity while multiple people are speaking	0.64	0.59

## Discussion

Standard video recording has been used in various clinical environments as it can easily provide informative feedback on overall team dynamics, individual performance, and patient care. Some clinical sites use video recordings to perform debriefings [17], while others may use video recordings to enhance detailed surgical procedures [18]. However, most of these videos have been captured with a standard 2D view camera, rather than a camera that features an entire panoramic view. With sweeping advances in camera technology, there have been limited studies on the efficacy of comparing SVCs to a 360° video camera, especially in neonatal resuscitation settings. This pilot study showed that 360V is a superior method for reviewing neonatal resuscitation simulations compared to two-view SVC.

Camera recordings using the 360V were rated to have improved visibility in comparison to two-view SVC cameras in critical neonatal resuscitation steps such as the enactment of team roles in PPV mask ventilation, endotracheal intubation, chest compressions, and many more. Audibility using 360V was also superior in capturing verbal communication between team members, ensuring closed-loop communication, distinguishing who is speaking, and maintaining clarity when multiple team members are talking. Therefore, we support our hypothesis that using 360V improves the visibility and audibility of neonatal resuscitation steps when compared to a two-view SVC.

Although there are clear benefits to 360V, a component that was superior in the two-view SVC included the lack of motion sickness. It is possible that the reviewers may have been more prone to motion sickness than the public; regardless, this suggests that the traditional two-view SVC provides a more stable viewing experience, reducing discomfort for reviewers. Further studies with more reviewer participants are necessary to understand the indication of this consequence to the overall benefits of 360V use.

This pilot study highlights the enhanced visibility and audibility of the 360V, yet it is worth exploring why some elements were better visualized while others showed no difference. While difficult to measure objectively, these differences may be influenced by recording quality, camera placement, and field of view. The 360V’s unobstructed, panoramic 4000-pixel resolution may have improved the visibility of large-motion tasks such as PPV, mask ventilation, and endotracheal intubation. However, more subtle tasks, such as ETCO_2_ confirmation, which require close-up visualization, were not consistently captured by either SVC or 360V. 

Video recording has been applied to various clinical and educational settings as it provides the opportunity for learners to reflect on events as portrayed rather than relying on faulty recall of the events [12]. Additional benefits of video-assisted debriefing include allowing learners to observe their own performance and review communication and dynamic teamwork from the perspective of a third party [12]. From this study, we add that improving the quality of the recordings by increasing visibility and audibility will allow learners and viewers to receive higher-quality feedback and informative analysis. One can imagine how improved visibility and audibility of 360V recordings can further enhance the quality and rate of experiential learning by targeting one’s own technical skills, communication habits, and overall team dynamics.

This study has some limitations. First, the study had a small sample size. We could not calculate the power needed to detect a difference between recording methods a priori because baseline resuscitation data using these devices was unavailable. This puts our results at risk for type II bias. Second, given the small number of observations, our analyses do not account for clustering by either reviewer or scenario. Third, this is a single-center study, which limits the generalizability of our results. Fourth, we did not include ratings of visibility or audibility from a direct observer of the simulation. This was based on prior studies showing variable interpretations of events by direct observers [19]. Finally, the locations of the SVC used in this study may not be the optimal placement for recording neonatal resuscitations. For example, placing the camera in different locations within the room may have yielded different results. Further studies comparing various locations of SVC and 360V are warranted.

## Conclusions

Average global visualization and audibility were improved using the 360V compared to two-view SVC. Therefore, 360V is a superior method for reviewing neonatal resuscitation simulations compared to two-view SVC. While this study focuses on the use of cameras in neonatal resuscitation simulations, the implications of 360V recording and its added benefits can be easily applied to other settings where improved visibility and audibility of individual and team performance are critical, such as surgical operating rooms or emergency resuscitation rooms. It can also be applied in medical school curricula, such as the Objective Structured Clinical Examination (OSCE), where students see standardized patients and are graded on their performance. However, further studies need to be conducted to assess the versatility of applications with the 360V and their true benefits in other clinical and non-medical spaces.
